# Supplementary material for: Genome-Wide Identification and Expression Analyses of the Thaumatin-Like Protein Gene Family in Tetragonia tetragonoides (Pall.) Kuntze Reveal Their Functions in Abiotic Stress Responses
Source: Plants (Basel). 2024 Aug 23;13(17):2355. doi: 10.3390/plants13172355 (PMC11397343; doi:10.3390/plants13172355)
Supplement: Supplementary file 1 [file plants-13-02355-s001.zip › plants-3145194-conversion final-Supplementary material.pdf]

Supplementary material

Figure S1 The pfam domain diagrams of 37 TtTLPs identified on the InterPro website (http://pfam.xfam.org/). The green representative domain showed the exact location and size of THAUMATIN_2 domains in every TtTLP protein. The protein length (amino acid numbers) of all TtTLPs were labeled.

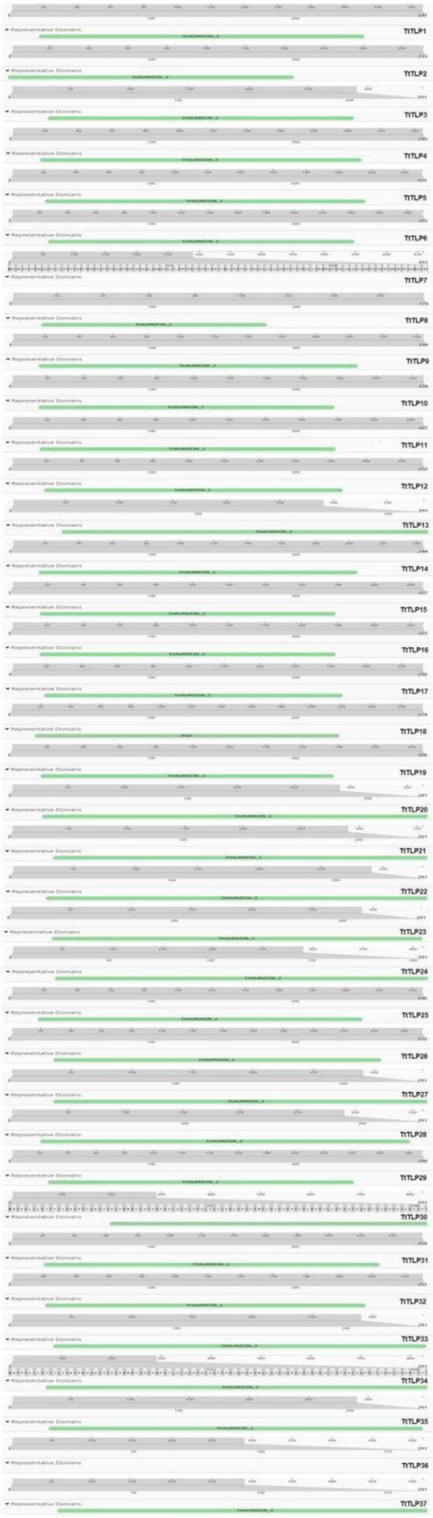

Table S1 Primer lists used in this study and *TtTLP* family sequence information.

Table S1.xlsx

Table S2 *TtTLP* family duplication patterns and Ka and Ks values. The gene replication modes include these major patterns: WGD (whole genome duplication), TD (tandem duplication), PD (proximal duplication), TRD (transposed duplication), DD (dispersed duplication), and SL (single). The Ka/Ks ratio of more than 1 (Ka/Ks > 1) suggests positive (non-purifying), and the ratio less than 1 (Ka/Ks < 1) indicates negative (purifying) selection pressure. The ratio equal to 1 (Ka/Ks = 1) indicates neutral selection.

Table S2.xlsx

Table S3 *Cis*-acting elements (CEs) identified in the promoter regions (ATG_upstream 2000 bp) and the numbers of all *TtTLP*s promoters' CEs.

Table S3.xlsx

Table S4 FPKM values of *TtTLP*s for the RNA-seq assay of *Tetragonia tetragonoides* tissues in this study. These values are all associated with the heat maps in this manuscript, including figures 6 and 7.

Table S4.xlsx
